# Long Term Control of Scabies Fifteen Years after an Intensive Treatment Programme

**DOI:** 10.1371/journal.pntd.0004246

**Published:** 2015-12-01

**Authors:** Michael Marks, Betty Taotao-Wini, Lorraine Satorara, Daniel Engelman, Titus Nasi, David C. Mabey, Andrew C. Steer

**Affiliations:** 1 Clinical Research Department, Faculty of Infectious and Tropical Diseases, London School of Hygiene & Tropical Medicine, London, United Kingdom; 2 Hospital for Tropical Diseases, University College London Hospitals NHS Trust, London, United Kingdom; 3 Department of Paediatrics, National Referral Hospital, Ministry of Health and Medical Services, Honiara, Solomon Islands; 4 National Health Training Research Institute, Ministry of Health and Medical Services, Honiara, Solomon Islands; 5 Centre for International Child Health, University of Melbourne, Melbourne, Victoria, Australia; 6 Department of General Medicine, Royal Children’s Hospital, Melbourne, Victoria, Australia; 7 Group A Streptococcal Research Group, Murdoch Childrens Research Institute, Melbourne, Victoria, Australia; University of California San Diego School of Medicine, UNITED STATES

## Abstract

**Introduction:**

Scabies is a major public health problem in the Pacific and is associated with an increased risk of bacterial skin infections, glomerulonephritis and rheumatic fever. Mass drug administration with ivermectin is a promising strategy for the control of scabies. Mass treatment with ivermectin followed by active case finding was conducted in five communities in the Solomon Islands between 1997 and 2000 and resulted in a significant reduction in the prevalence of both scabies and bacterial skin infections.

**Methods:**

We conducted a prospective follow-up study of the communities where the original scabies control programme had been undertaken. All residents underwent a standardised examination for the detection of scabies and impetigo.

**Results:**

Three hundred and thirty eight residents were examined, representing 69% of the total population of the five communities. Only 1 case of scabies was found, in an adult who had recently returned from the mainland. The prevalence of active impetigo was 8.8% overall and 12.4% in children aged 12 years or less.

**Discussion:**

We found an extremely low prevalence of scabies 15 years after the cessation of a scabies control programme. The prevalence of impetigo had also declined further since the end of the control programme. Our results suggest that a combination of mass treatment with ivermectin and intensive active case finding may result in long term control of scabies. Larger scale studies and integration with other neglected tropical disease control programmes should be priorities for scabies control efforts.

## Author Summary

Scabies is a parasitic infection caused by the mite *Sarcoptes scabiei*. Scabies is a risk factor for bacterial skin infections and as a result of these secondary infections, individuals with scabies are also at risk of kidney disease and possibly rheumatic heart disease. Traditionally the management of scabies has involved treating individuals and close contacts with topical medicines. Although this normally cures the patient, individual treatment does not have any impact on the overall amount of scabies in a community. There is now significant interest in trying mass drug administration (MDA) against scabies using ivermectin, a tablet which is a very effective treatment. A key question for scabies control programmes is how long the beneficial effect of MDA lasts. In this study we provide fifteen year follow-up on one of the first communities in the world to ever receive ivermectin MDA for the control of scabies. These communities had received intensive scabies control between 1997 and 2000 but then no further MDA or surveillance. We found that the amount of scabies had remained extremely low following the treatment programme and that bacterial skin infections had also remained low. This data suggests that long term control of scabies can be achieved with an initial intensive control programme.

## Introduction

Scabies, caused by infestation with the *Sarcoptes scabiei* mite, remains a major public health problem in developing countries worldwide[1]. In addition to the direct manifestations of infestation, scabies is associated with an increased risk of bacterial skin infections due to *Staphylococcus aureus* and *Streptocococcus pyogenes*[2] and. as a result, with both glomerulonephritis and possibly rheumatic heart disease[1,3]. Treatment of scabies also results in a reduction of secondary skin infections [4], even in the absence of specific anti-bacterial therapy, and the benefits of treatment might therefore extend well beyond scabies itself.

Worldwide the highest burden of scabies is reported from the Pacific[5] with a prevalence as high as 30–40% reported in some studies[4,6]. Alongside the substantial burden of scabies, a high prevalence of impetigo and secondary bacterial skin infections is also reported in these settings. Most studies have reported that the prevalence of both scabies and secondary bacterial infection is higher in children than in adults[5].

In endemic areas with high prevalence mass treatment has been advocated as a promising intervention to control scabies at a population level[7,8]. It is well recognised that simultaneous treatment of all household members is required to prevent re-infestation from occurring. The treatment of patients presenting to health care facilities with scabies does not have a long term impact on the community prevalence of scabies, presumably because re-infestation is common in endemic settings. A study evaluating the impact of mass treatment with topical permethrin conducted in Panama demonstrated a substantial reduction in the prevalence of scabies[8]. The control programme was interrupted by the subsequent American invasion of Panama, resulting in the breakdown of the control programme and a resurgence of scabies.

The use of the oral agent ivermectin, has been advocated as an alternative to topical treatments for scabies [9,10]. A number of studies have shown that ivermectin is equivalent or superior to commonly used topical treatments such as benzyl benzoate[11–13]. Ivermectin has been used as part of mass drug administration (MDA) programmes for the elimination of lymphatic filariasis and onchocerciasis, and an indirect assessment from Zanzibar showed a marked decrease in incident cases of scabies seen in clinics was reported[14]. Two small scale assessments of MDA with ivermectin, conducted in Papua New Guinea[15] and the Solomon Islands[4], have both demonstrated a significant reduction in the prevalence of scabies, an accompanying reduction in the prevalence of secondary bacterial skin infections. These studies have also shown some evidence for a reduction in the prevalence of post-streptococcal glomerulonephritis. A further study in Fiji which compared MDA with either ivermectin or topical benzyl-benzoate showed that both strategies effectively reduced the prevalence of scabies at a community level[7].

**Fig 1:**
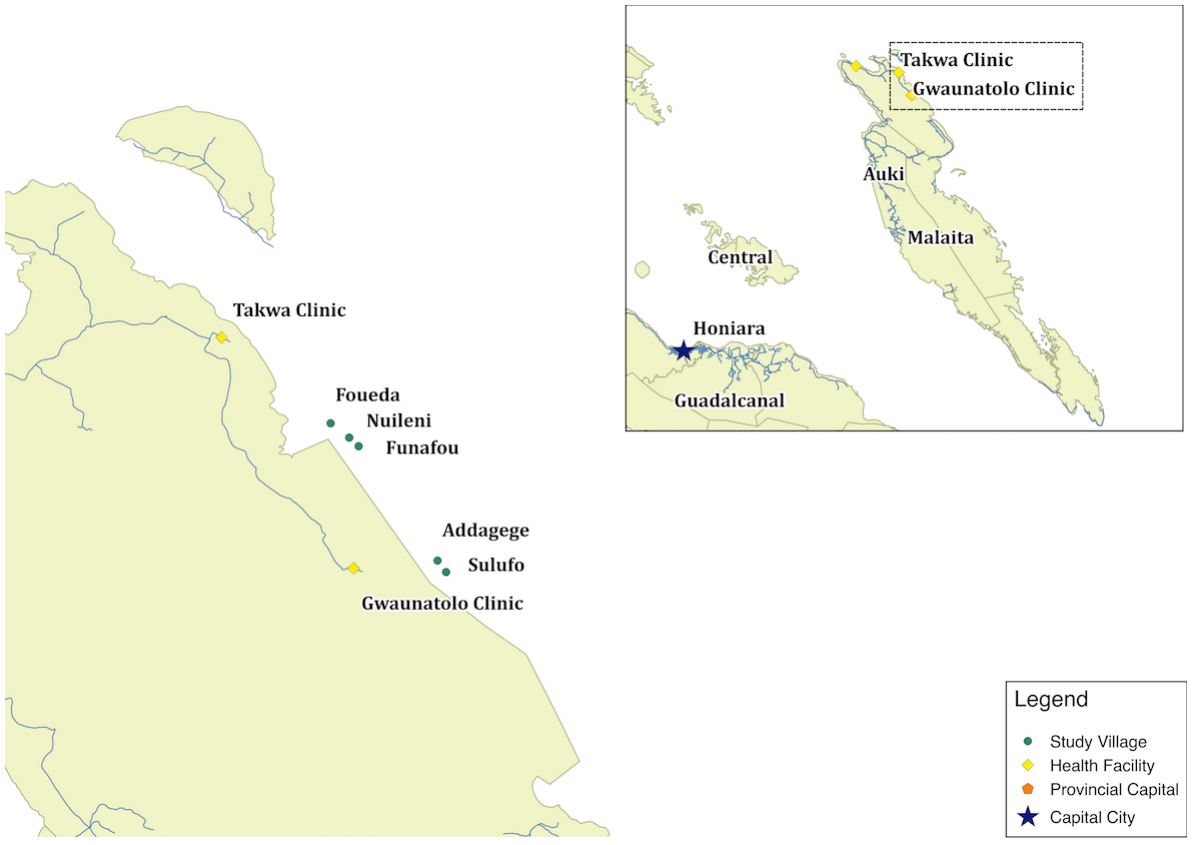
Location of villages. The map highlights the position of the five villages included in the original scabies control programme. Local health clinics and the provincial capital Auki are also shown.

The first attempt at MDA for scabies control using ivermectin was conducted between 1997 and 2000 in five communities in the Lau Lagoon in the Solomon Islands. The selected communities, Addagege, Foueda, Funafou, Nuileni and Sulufo lived on isolated artificial islands off the mainland of Malaita (Fig 1). Whilst residents travel to and from the mainland to farm the population is relatively isolated from the mainland population. The methodology of the programme has been described in detail elsewhere [4]. Briefly, a step-wedge design was used. There was an initial round of community mass treatment of all the inhabitants of each community with a single dose of ivermectin (200μg/kg), or permethrin where ivermectin was contraindicated. Residents who had been absent at the time of the initial mass treatment were offered treatment on their return, irrespective of whether they clinically were found to have scabies or not. The initial mass treatment was followed by active case finding surveys conducted three times a year by local nursing staff. Individuals found to have scabies at a follow-up visit were retreated along with their household contacts. The prevalence of scabies in children fell from 25% at the start of the study to 0.7% at the final visit. There was an accompanying decline in the prevalence of impetigo from 40% to 22%. The prevalence of scabies in adults fell from approximately 20% at the start of the study to 0.8% at the final visit. Since the cessation of the study in 2000 no further active control measures have been undertaken in these communities. We conducted a cross-sectional survey to establish the prevalence of scabies and impetigo 15 years after the cessation of the scabies control programme in these communities.

## Methods

### Participant Recruitment

The follow-up survey was undertaken in August 2015. Verbal consent was obtained from village chiefs to conduct the survey. We then undertook a house-to-house survey with village elders to obtain an accurate headcount of individuals currently living in each community. All residents of the five communities were invited to participate in the study.

### Ethics Statement

Written informed consent was obtained from participants by staff fluent in local dialect. For children <18 years, written consent was obtained from an adult parent or guardian and verbal assent was obtained from the child. Ethical approval for the study was granted by the London School of Hygiene & Tropical Medicine (LSHTM-9146) and the Solomon Islands National Health Ethics Research Committee (HRC-15/14).

### Data Collection

For each participant we collected demographic data including age and gender, as well as data on household size and number of individuals sharing a single room as measures of overcrowding. We collected data on whether any household contacts had been diagnosed with scabies. All participants were then examined by an experienced consultant paediatrician (BTW). We recorded the presence or absence of all skin lesions, their distribution and whether they were consistent with scabies, impetigo or an alternative diagnosis. The number of scabies lesions was classified as ≤10, 11 to 49 or ≥50. Lesions of impetigo were classified as either active (moist, purulent or crusted lesions) or healed (flat/dry lesions with no crust). The number of impetigo lesions was classified as ≤5, 6 to 10, 11 to 49 or ≥50. All data was collected directly on to Android smartphones using the ODK software package[16].

### Statistical Analysis

As the aim of the study was to examine the resident population of each community no formal sample size calculation was undertaken. We estimated the prevalence of scabies and impetigo stratified by age and gender with exact confidence intervals based on the binomial distribution. Logistic regression was used to calculate odds ratios associated with scabies and impetigo. For the purpose of analysis we stratified age into young children (age ≤12), adolescents (aged 13–18) and adults (≥18). A cut-off of twelve years was selected for defining young children as this was the age cut-off used to define children in the original scabies control programme study. Household size was classified as ≤5 or >5 residents, which is the national average household size based on the 2009 census[17]. We classified the number of individuals sharing a room as 1, 2 or ≥3. All analyses were conducted using STATA 13.1 (Statcorp, Texas, USA). The paper is reported in line with the STROBE checklist for observational studies[18] (S1 Checklist).

## Results

According to the initial house-to-house survey 561 individuals were reported to be currently resident in the five communities. In total 388 individuals (69.2%), from 121 households were present during the survey period. All individuals present at the time of the survey consented to participate in the current study. The median age was 15 years (IQR 7–38) and 221 participants were female (57%). The median reported household size was 4 residents (IQR 3–6) and the median number of examined individuals per household was 3 (IQR 2–4). A majority of individuals slept in the same room as 3 or more individuals (n = 196, 50.5%) (Table 1).

**Table 1:** Demographic characteristics.

Variable	n (%)
Village	
Addagege	32 (8.2%)
Foueda	46 (11.9%)
Funafou	95 (24.5%)
Nuileni	60 (15.5%)
Sulofu	155 (39.9%)
Gender	
Male	167 (43.0%)
Female	221 (57.0%)
Age (years)	
≤12	169 (43.7%)
13–18	44 (11.3%)
≥19	174 (45.0%)
Household Size	
≤5	239 (61.6%)
>5	149 (38.4%)
Individuals sleeping in same room (n)	
1	59 (15.2%)
2	133 (34.3%)
≥3	196 (50.5%)

Scabies was diagnosed in one individual (0.26%, 95% CI 0.0–1.4%). Twelve individuals (3.1%) from seven households reported a household contact had been diagnosed with scabies in the last twelve months.

Active impetigo was diagnosed in 34 individuals (8.8%, 95% CI 6.1–12.0%). Most patients with impetigo (n = 31, 91.2%) had a small number of lesions (≤5). Active impetigo was more common in children aged twelve years or less (OR 2.60, p < 0.021) compared to adults. Impetigo was also more common in children aged 13–18 years than in adults, and in males than in females, although these differences were not statistically significant (Table 2). Household size was not associated with the risk of active impetigo (p = 0.44). The risk of active impetigo increased with the number of inhabitants sharing a room but this association was not statistically significant (p = 0.19) (Table 2). The prevalence of impetigo did not differ significantly between villages (p = 0.37).

**Table 2:** Risk factors for impetigo.

Variable	Prevalence	Odds Ratio (95% CI)
Gender		
Male	12.0%	2.0 (1.0–4.1)
Female	6.3%	1
Age (years)		
≤12	12.4%	2.6 (1.2–5.9)
13–18	9.1%	1.8 (0.5–6.3)
≥19	5.2%	1
Household Size		
≤5	9.6%	1
>5	7.4%	0.8 (0.3–1.6)
Individuals sleeping in same room (n)		
1	3.4%	1
2	10.5%	3.4 (0.7–15.3)
≥3	9.2%	2.9 (0.7–12.8)

Evidence of healed impetigo was found in 119 individuals (30.7%, 95% CI 26.1–35.5%). Other commonly noted skin lesions included molluscum contagiosum (n = 15, 3.9%), ringworm (n = 9, 2.3%) and warts (n = 8, 2.1%).

## Discussion

In this study we found an extremely low prevalence of scabies fifteen years after the cessation of an intensive control programme in five communities in the Solomon Islands. Only one case of scabies was found after examining almost four hundred participants. On questioning, this individual had recently returned from spending several nights in a village on the mainland, and therefore it is possible therefore that this case represents an imported infection. The prevalence of scabies found in this follow-up study is similar to that at the end of the original control programme in 2000 and suggests that the intervention undertaken has resulted in a sustained reduction in the prevalence of scabies on these islands. Of interest, the original study used only a single dose of ivermectin[4], rather than the two dose regime that is now recommended[19]. It is likely that the frequent follow-up and retreatment undertaken in the control programme mitigated the effect of a reduced initial dose and contributed to the sustained reduction in scabies prevalence observed.

Alongside this extremely low prevalence of scabies we found a relatively low prevalence of impetigo. The prevalence of impetigo in children aged 12 or less at the beginning of the scabies control programme had been 40% but declined to 21% by the end of the intervention. In our survey this had declined still further to 12%, suggesting that the long term reduction in scabies has had a continuing effect in reducing, although not eliminating, bacterial skin infections.

In all five communities the study team met residents who recalled the original scabies control intervention. Community leaders all reported that since the completion of the control programme, scabies was no longer a significant health problem in the villages. Nurses in the local clinics serving the communities offered similar evidence; staff at both clinics reported that scabies cases were seen rarely from the study communities but that cases continued to be seen from villages on the mainland of the province. Although anecdotal, this evidence is in keeping with the low prevalence of scabies infestation found on examination and the extremely small number of individuals who reported a household contact who had been diagnosed with scabies.

Our study has a number of weaknesses, most notably the long period since the last survey was conducted. It is possible that secular trends such as a reduction in overcrowding or improvements in access to care have contributed in part to the low prevalence of scabies in our current survey. However, markers of broader secular development are largely unchanged in the Solomon Islands since the original scabies intervention was completed. For example there has been no increase in mean years of education or GDP per capita between 2000 and 2012 and the UNDP Human development index for the Solomon Islands has not changed substantially from the time of the original scabies intervention (HDI in year 2,000 = 0.475) to the time of the current study (HDI in year 2013 = 0.491)[20]. Scabies has been reported to be cyclical in nature in some countries[21], but this phenomenon is not observed in many tropical countries[21,22]. The ideal study design would have involved a control group of communities which did not receive MDA and collecting data on scabies and impetigo both at baseline (1997–2000) and at the time of this follow-up survey (2015). As there were no control communities included in the original survey such a study design was not possible. However, surveys conducted in two other provinces of the Solomon Islands in the last year have observed prevalence of scabies that is similar to that found in the study communities before MDA was conducted (Andrew Steer—Personal Communication). Taken together, these data add weight to the argument that the low prevalence of scabies and impetigo found in the current survey is attributable to the original intervention and not to secular or cyclical trends in scabies prevalence.

We were not able to examine all individuals who were resident on the islands. Overall we examined close to 70% of the population and the most frequently absent group of individuals were adult males. As scabies is predominantly found in children, it seems unlikely that the exclusion of older males has resulted in a significant under estimate of the prevalence of scabies. At the time of the previous survey the population of the five communities was officially recorded at 1,558 but only half of these individuals (approximately 750 people) were actually reported to be resident on the islands. At the time of the current survey the resident population of the communities had decreased slightly to 561 although officially the population of Sulofu alone is close to 1,000. The majority of the children enrolled in this study were born after the cessation of the scabies control programme and it seems unlikely therefore that the extremely low prevalence of scabies is explained by herd immunity. Finally it is possible that examination missed cases of scabies occurring in unexposed areas of the skin such as genitals or breasts. We used the same exam exposure as was used in the original study[4] so it seems unlikely that this contributed significantly to an under estimate of the prevalence of scabies.

Our data add to increasing evidence that community MDA with ivermectin is an effective intervention for the control of scabies. The control strategy used in these communities included additional interventions following the initial round of mass treatment. This is similar to the World Health Organization strategy for the eradication of yaws[23], which also comprises an initial round of community MDA, with azithromycin, followed by 6 monthly intensive case finding and retreatment. Given the considerable epidemiological overlap between the two diseases, especially in the Pacific[24], and the fact that a dermatological examination would allow simultaneous surveillance for both diseases, an integrated approach should be considered for these two neglected tropical diseases. Azithromycin and ivermectin have been safely co-administered as part of a study on integrated treatment for trachoma and lymphatic filariasis [25] and larger scale studies would be of benefit in facilitating integrated control programmes.

The extremely low prevalence of scabies found in this study, despite the cessation of the scabies control programme for more than 15 years, suggests that long-term disease control is a realistic possibility. Large-scale studies of MDA and developing an evidence base to support integration with other NTD control programmes should be priorities for the global control of scabies worldwide.

## Supporting Information

S1 Checklist Strobe Checklist. (DOCX)

S1 File Supplementary Data File. (CSV)
